# Higher serum homocysteine levels are associated with an increased risk of hemorrhagic transformation in patients with acute ischemic stroke

**DOI:** 10.1186/s12883-023-03137-2

**Published:** 2023-03-11

**Authors:** Qian Wu, Junfeng Liu, Yanan Wang, Yajun Cheng, Ming Liu

**Affiliations:** Center of Cerebrovascular Diseases, Department of Neurology, West China Hospital, Sichuan University, No. 37, Guo Xue Xiang, 610041 Chengdu, Sichuan Province China

**Keywords:** Homocysteine, Hemorrhagic transformation, Parenchyma hemorrhage, Acute ischemic stroke

## Abstract

**Background:**

Hemorrhagic transformation (HT) is a common complication of acute ischemic stroke (AIS), and may develop into parenchyma hemorrhage (PH). We aimed to investigate the association between serum homocysteine levels and HT as well as PH in all AIS patients, and in those with and without thrombolysis by subgroup analysis.

**Methods:**

AIS patients who were admitted within 24 h after onset were enrolled and categorized into the higher homocysteine level group (≥ 15.5 µmol/L) and the lower homocysteine level group (< 15.5 µmol/L). HT was determined by a second round of brain imaging within 7 days during hospitalization, and PH was defined as hematoma in the ischemic parenchyma. Multivariate logistic regression was used to investigate the associations between serum homocysteine levels and HT and PH, respectively.

**Results:**

Of the 427 included patients (mean age 67.35 years, 60.0% males), 56 (13.11%) developed HT and 28 (6.56%) had PH. Serum homocysteine levels were significantly associated with HT (adjusted OR 1.029, 95%CI 1.003–1.055) and PH (adjusted OR 1.041, 95%CI 1.013–1.070). The higher homocysteine group was more likely to have HT (adjusted OR 1.902, 95% CI 1.022–3.539) and PH (adjusted OR 3.073, 95% CI 1.327–7.120) than the lower homocysteine group. Subgroup analysis of patients without thrombolysis also showed the significant differences in HT (adjusted OR 2.064, 95% CI 1.043–4.082) and PH (adjusted OR 2.926, 95% CI 1.196–7.156) between the two groups.

**Conclusion:**

Higher serum homocysteine levels are associated with an increased risk of HT and PH in AIS patients, especially in those without thrombolysis. Monitoring the serum homocysteine may be conducive to determining individuals at a high risk of HT.

**Supplementary Information:**

The online version contains supplementary material available at 10.1186/s12883-023-03137-2.

## Background

Hemorrhagic transformation (HT) is the secondary bleeding that occurs in the ischemic area or distant brain tissues after recovery of blood flow and recanalization of occluded vessels and is also a natural course of acute ischemic stroke (AIS) [1]. It is usually associated with poor prognosis [2]. HT may cause delayed administration of antithrombotic therapies in AIS patients [3, 4]. Use of effective treatments (thrombolysis or thrombectomy) seems to be in a dilemma partly due to the excessive fear of HT and poor prognosis [5]. HT is classified into hemorrhagic infarction and parenchymal hematoma (PH) according to the radiographic features [6]. Patients with PH have a higher risk of death and disability compared to those without PH [7, 8]. Therefore, investigating key factors associated with HT and PH may be conducive to identifying individuals at a high risk of hemorrhage and selecting appropriate therapeutic interventions.

In recent years, interest in the role of homocysteine in ischemic stroke and related pathophysiological process, including blood-brain barrier (BBB) damage [9, 10], inflammation response [11] and poor outcomes [12–14] has increased. Disruption of the BBB plays a crucial role in the occurrence and development of HT after cerebral infarction [1]. It is likely that homocysteine is associated with an increased risk of HT. At present, some studies have explored the relationship between serum homocysteine levels and HT in AIS patients receiving thrombolysis [15, 16], but their conclusions were not consistent. Furthermore, the vast majority of ischemic stroke patients do not undergo thrombolysis [17], and such population were not involved in these studies. Therefore, the association between serum homocysteine levels and HT in patients with AIS remains unclear.

In the present study, we aimed to investigate the association between serum homocysteine levels and HT, as well as PH, in all AIS patients, and in those with and without thrombolysis by subgroup analysis.

## Methods

### Study design and participants

AIS patients admitted within 24 h of stroke onset were prospectively enrolled from Chengdu Stroke Registry Database of the West China Hospital [18] from January 2015 to September 2018. The diagnosis of AIS was based on World Health Organization criteria [19] and confirmed by head computed tomography (CT) scan or magnetic resonance imaging (MRI). This study was conducted in accordance with the ethical principles of the Declaration of Helsinki (1964) [20]. Patients were eligible for the study if they [1] were over 18 years old; [2] had no stroke occurrence or head trauma within the previous three months; [3] underwent head CT/MRI on admission; [4] had serum homocysteine measured after admission. Patients were excluded if they [1] had hemorrhage on initial CT/MRI; [2] did not undergo a second round of CT/MRI during the hospitalization; [3] had an occurrence of HT before the serum homocysteine test; [4] had an infectious, inflammation disease, severe liver and renal dysfunction, hematological disease, or malnutrition; [5] refused to participate in the study.

### Data collection

Baseline information and clinical characteristics of patients were collected and recorded using a case report form including age, sex, blood pressure on admission, National Institutes of Health Stroke Scale (NIHSS) score on admission, vascular risk factors (hypertension, diabetes mellitus, hyperlipidemia, atrial fibrillation, current smoking and alcohol consumption), previous history of cardiovascular events (myocardial infarction, transient ischemic attack, ischemic and hemorrhagic stroke), medication after onset (thrombolysis, thrombectomy, anticoagulants, antiplatelet drugs and statins), results of laboratory tests (blood glucose and creatine on admission), and interval time from stroke onset to admission. According to the criteria of the Trial of Org 10,172 in Acute Stroke Treatment (TOAST)[21], at least two neurologists independently classified the stroke type in each patient into one of the following categories: large-artery atherosclerosis, small-vessel occlusion, cardio-embolism, stroke of other determined etiology and stroke of undetermined etiology. The serum homocysteine levels (µmol/L) were measured by clinical laboratory technicians within 48 h after admission and the results were acquired directly from the medical records. Time points of blood samples collection were also recorded.

### CT and MRI scans and definition of HT

All patients underwent brain CT/MRI scanning within 24 h of admission followed by a second scheduled MRI within 7 days during hospitalization or a subsequent CT scan immediately whenever suspicion of an intracerebral hemorrhage occurred, such as sudden headache or abrupt neurological deterioration. CT scanning was performed on a 64-section scanner (Aquilion) with 5-mm slice thickness. MRI was performed using a 3-T Siemens Magnetom Trio scanner with 5 mm slice thickness, including sequences of T1-weighted (TR, 1600 ms; TE, 8.6ms), T2-weighted (TR, 4500 ms; TE, 105ms) and fluid-attenuated inversion recovery (FLAIR) images (TR, 6000 ms; TE,100ms). HT was defined as intracerebral hemorrhage which was not detected by CT/MRI images on admission but by a subsequent CT/MRI scan [22], which is the same definition in our previous report [23]. In accordance with the recommendations of European Cooperative Acute Stroke Study (ECASS)[24], HT was radiographically classified as hemorrhagic infarction or parenchyma hemorrhage (PH). In the present study, HT was separately determined and classified by two experienced neurologists blinded to other clinical information. A third neurologist was consulted to solve any divergence of opinion.

### Statistical analysis

Continuous variables were reported as mean ± standard deviation or as median (interquartile range, IQR). Categorical variables were reported as number (percentages). Inter-group differences in continuous variables were assessed using Student’s t-test or Mann-Whitney U test. Differences in categorical variables were analyzed by chi-square test or Fisher’s exact test. To investigate the dose-response associations between serum homocysteine (µmol/L) levels and risk of HT and PH, respectively, we performed restricted cubic spline models fitted for multivariable logistic regression models with 4 knots at the 5th, 35th, 65th, and 95th percentiles of homocysteine. Patients were categorized into the higher homocysteine level group (≥ 15.5 µmol/L) and the lower homocysteine level group (< 15.5 µmol/L), as the restricted cubic spline models revealed increased odds ratio of both HT and PH above this level. Multivariate logistic analysis was used to determine associations between serum homocysteine levels and HT and PH, respectively. In model 1, we adjusted for sex and age. In model 2, we adjusted for sex, age, NIHSS on admission, atrial fibrillation, TOAST classification of stroke etiology, and interval time from admission to blood sampling. Odds ratios (ORs) and 95% confidence intervals (CIs) were reported both in univariate and multivariate analysis. Stratified logistic regression models were used to perform subgroup analysis (age, sex, NIHSS on admission, hypertension, atrial fibrillation, current smoking, medical treatments after onset including thrombolysis, thrombectomy and anticoagulants). The significance of interaction (p for interaction) between serum homocysteine and stratified factors was tested using the likelihood ratio test.

All data were analyzed using SPSS software (version 23.0; IBM, Chicago, IL, USA) and STATA 16.0 (Corporation, College Station, TX). A threshold of two-side P value < 0.05 was identified as statistically significant.

## Results

### Baseline characteristics

**Fig. 1:**
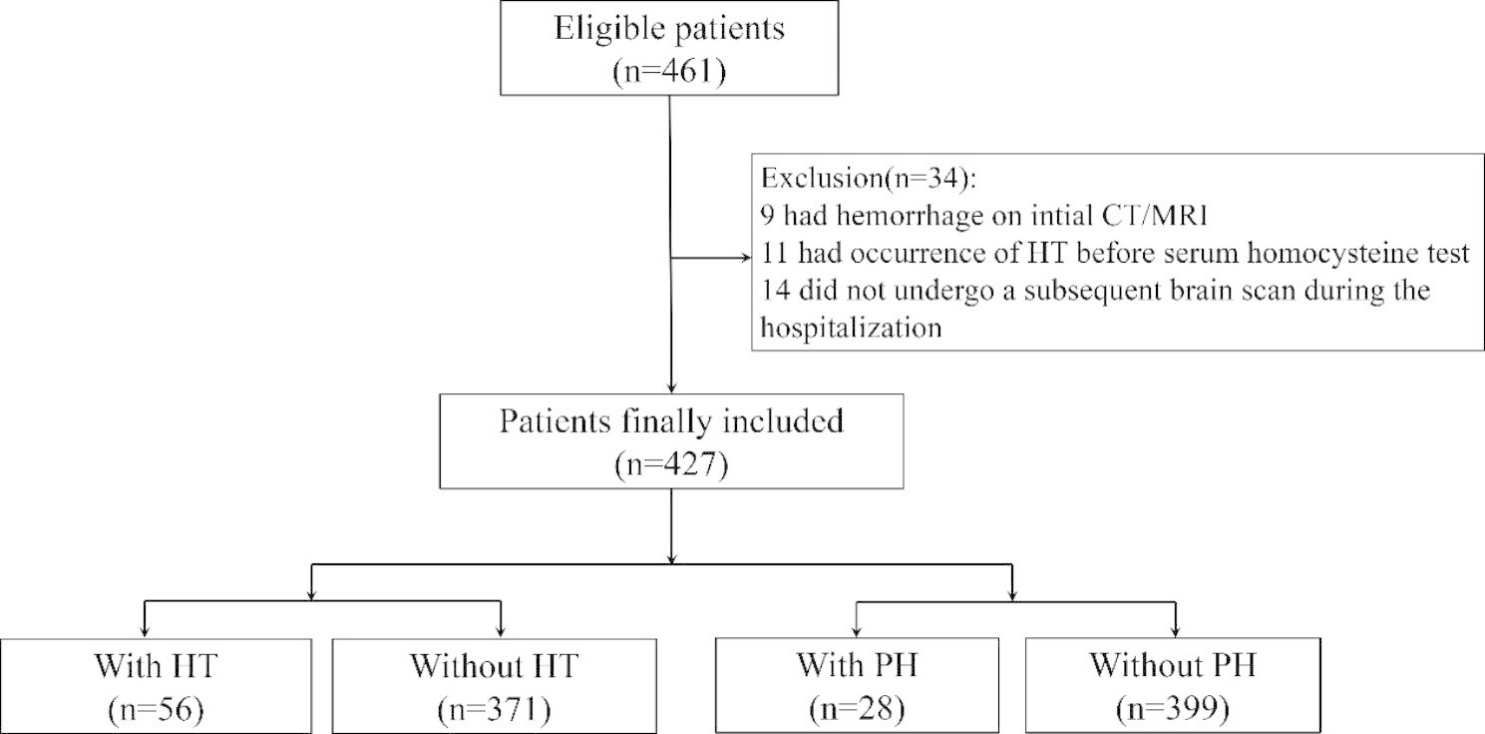
The flow chart of the study patients. CT: computed tomography; MRI: magnetic resonance imaging; HT: hemorrhagic transformation; PH: parenchymal hematoma

A total of 1156 participants with AIS were enrolled from Chengdu Stroke Registry. As the flow chart of the study (Fig. 1) showed, 461 patients with complete clinical data were initially evaluated. After excluding 34 patients (9 had hemorrhage on initial CT/MRI, 11 had an occurrence of HT before serum homocysteine test and 14 did not undergo a subsequent brain scan during the hospitalization), 427 patients (60.0% males, mean age 67.35 ± 13.85 years) were included in the final analysis. The baseline characteristics of the patients are presented in Table 1. Among the included patients, 13.11% (56/427) developed HT and 6.56% (28/427) had PH. The median NIHSS score on admission was 6 (IQR, 3–13). The median level of homocysteine in total was 13.5µmol/L (IQR, 10.9–16.8 µmol/L). The median interval time from stroke onset to admission was 7.0 (IQR, 3.5–24.0) hours.

**Table 1:** Baseline characteristics of included patients grouped by HT or PH

Variables	Total (n=427)	HT			PH		
		Yes (n=56)	No (n=371)	P value	Yes (n=28)	No (n=399)	P value
Sex, male	256 (60.0)	29 (51.8)	227 (61.2)	0.181	16 (57.1)	240 (60.2)	0.754
Age, years	67.35±13.85	70.20±13.17	66.92±13.91	0.099	70.89±11.73	67.10±13.96	0.112
Systolic BP on admission, mmHg	147.71±23.94	145.75±23.25	148.01±24.06	0.512	147.89±23.38	147.70±24.01	0.967
Diastolic BP on admission, mmHg	85.47±14.94	87.50±17.95	85.16±14.44	0.355	90.39±19.54	85.12±14.54	0.071
NIHSS on admission	6.0 (3.0-13.0)	11.5 (8.0-15.8)	5.0 (2.0-12.0)	<0.001	12.0 (8.3-14.8)	5.0 (2.0-12.0)	<0.001
Vascular risk factors							
Hypertension	247 (57.8)	37 (66.1)	210 (56.6)	0.181	19 (67.9)	228 (57.1)	0.267
Diabetes mellitus	88 (20.6)	12 (21.4)	76 (20.5)	0.871	6 (21.4)	82 (20.6)	0.912
Hyperlipidemia	17 (4.0)	1 (1.8)	16(4.3)	0.711	0 (0.0)	17(4.3)	0.617
Atrial fibrillation	74 (17.3)	20 (35.7)	54 (14.6)	<0.001	11 (39.3)	63 (15.8)	0.004
Current smoking	160 (37.5)	20 (35.7)	140 (37.7)	0.771	11 (39.3)	149 (37.3)	0.837
Alcohol consumption	103 (24.1)	11 (19.6)	92 (24.8)	0.401	9 (32.1)	94 (23.6)	0.305
Previous history of cardiovascular events							
Myocardial infarction	11 (2.6)	0 (0.0)	11 (3.0)	0.373	0 (0.0)	11 (2.8)	1.000
Transient ischemic attack	9 (2.1)	0 (0.0)	9 (2.4)	0.614	0 (0.0)	9 (2.3)	1.000
Previous ischemic stroke	71 (16.6)	9 (16.1)	62 (16.7)	0.905	5 (17.9)	66 (16.5)	0.796
Previous hemorrhagic stroke	6 (1.4)	0 (0.0)	6 (1.6)	1.000	0 (0.0)	6 (1.5)	1.000
TOAST classification of stroke etiology				<0.001			0.003
Large-artery atherosclerosis	128 (30.0)	16(28.6)	112(30.2)		9(32.1)	119(29.8)	
Small-vessel occlusion	104 (24.4)	1(1.8)	103(27.8)		0(0.0)	104(26.1)	
Cardioembolism	116 (27.2)	26(46.4)	90(24.3)		13(46.4)	103(25.8)	
Other cause	12 (2.8)	1(1.8)	11(3.0)		1(3.6)	11(2.8)	
Undetermined etiology Stroke	67 (15.7)	12(21.4)	55(14.8)		5(17.9)	62(15.5)	
Homocysteine, umol/L	13.5(10.9-16.8)	15.0(11.5-17.9)	13.3(10.8-16.6)	0.048	16.5(12.5-21.1)	13.3(10.8-16.4)	0.008
Glucose on admission, mmol/L	7.97±3.36	7.96±2.09	7.97±3.51	0.986	8.26±2.40	7.95±3.42	0.635
Blood creatine on admission, μmol/L	80.36±42.42	79.81±37.36	80.44±43.18	0.917	84.15±43.01	80.09±42.42	0.625
Therapies after stroke onset							
Thrombolysis	44 (10.3)	9 (16.1)	35 (9.4)	0.154	3 (10.7)	41(10.3)	1.000
Thrombectomy	20 (4.7)	5 (8.9)	15 (4.1)	0.163	2 (7.1)	18 (4.5)	0.384
Anticoagulation	61 (14.3)	9 (16.1)	52 (14.0)	0.682	3 (10.7)	58 (14.5)	0.782
Antiplatelet	405 (94.8)	49(87.5)	356 (96.0)	0.016	23(82.1)	382 (95.7)	0.010
Statin	400 (94.6)	50 (89.3)	350 (95.4)	0.103	26 (92.9)	374 (94.7)	0.658
Interval time from stroke onset to admission, h	7.0(3.5-24.0)	5(4.0-19.8)	8.0(3.5-24.0)	0.110	5.5(4.0-22.8)	8.0(3.5-24.0)	0.481
Interval time from admission to blood sampling, h	34.5(18.6-44.1)	23.8(14.5-41.9)	35.2(19.1-44.2)	0.023	22.1(15.1-41.2)	34.8(18.9-44.1)	0.129

Values are n (%) or mean ± SD or median (IQR)

Abbreviation: HT, hemorrhagic transformation; PH, parenchyma hemorrhage; BP, blood pressure; NIHSS, National Institutes of Health Stroke Scale; TOAST classification of stroke etiology, the Trial of ORG10172 in the Acute Stroke Treatment

The demographic and clinical characteristics between the lower homocysteine level group and the higher homocysteine level group were compared (Table 2). Patients with higher serum homocysteine levels were older and more likely to be male and had a higher blood creatine level on admission (All P < 0.001). The proportions of hypertension, diabetes mellitus and atrial fibrillation were higher in the higher homocysteine level group compared with the lower homocysteine level group (all P < 0.05).

**Table 2:** Comparison of clinical characteristics between the lower and the higher homocysteine level group

Variables	Homocysteine level (μmol/L)		P value
	Lower group (<15.5 umol/L, n=285)	Higher group (≥15.5 umol/L, n=142)	
Sex, male	153(53.7)	103(72.5)	<0.001
Age, years	65.51±13.47	71.05±13.90	<0.001
Systolic BP on admission, mmHg	147.21±23.92	148.70±24.04	0.733
Diastolic BP on admission, mmHg	85.31±14.50	85.78±15.85	0.545
NIHSS on admission	5.0(2.5-12.0)	8.0(3.0-14.0)	0.018
Vascular risk factors			
Hypertension	148(51.9)	99(69.7)	<0.001
Diabetes mellitus	49(17.2)	39(27.5)	0.013
Hyperlipidemia	14(4.9)	3(2.1)	0.163
Atrial fibrillation	42(14.7)	32(22.5)	0.045
Current smoking	105(36.8)	55(38.7)	0.704
Alcohol consumption	64(22.5)	39(27.5)	0.254
Previous history of cardiovascular events			
Myocardial infarction	5(1.8)	6(4.2)	0.193
Transient ischemic attack	3(1.1)	6(4.2)	0.066
Previous ischemic stroke	43(15.1)	28(19.7)	0.226
Previous hemorrhagic stroke	2(0.7)	4(2.8)	0.098
TOAST classification of stroke etiology			0.820
Large-artery atherosclerosis	87(30.5)	41(28.9)	
Small-vessel occlusion	72(25.3)	32(22.5)	
Cardioembolism	73(25.6)	43(30.3)	
Other cause	9(3.2)	3(2.1)	
Undetermined etiology Stroke	44(15.4)	23(16.2)	
Glucose on admission, mmol/L	8.06±3.53	7.78±2.98	0.425
Blood creatine on admission, μmol/L	71.86±18.18	97.34±65.77	<0.001
Therapies after stroke onset			
Thrombolysis	31(10.9)	13(9.2)	0.581
Thrombectomy	12(4.2)	8(5.6)	0.517
Anticoagulation	41(14.4)	20(14.1)	0.933
Antiplatelet	276(96.8)	129(90.8)	0.008
Statin	269(95.7)	131(92.3)	0.137
Interval time from stroke onset to admission, h	7.0(4.0-24.0)	6.0(3.0-24.0)	0.568
Interval time from admission to blood sampling, h	18.1(13.0-21.1)	18.3(14.1-21.4)	0.771
HT	29(10.2)	27(19.0)	0.011
PH	11(3.9)	17(12.0)	0.001

Values are n (%) or mean ± SD or median (IQR)

Abbreviation: HT, hemorrhagic transformation; PH, parenchyma hemorrhage; BP, blood pressure; NIHSS, National Institutes of Health Stroke Scale; TOAST classification of stroke etiology, the Trial of ORG10172 in the Acute Stroke Treatment

### Association between serum homocysteine and HT

**Fig. 2:**
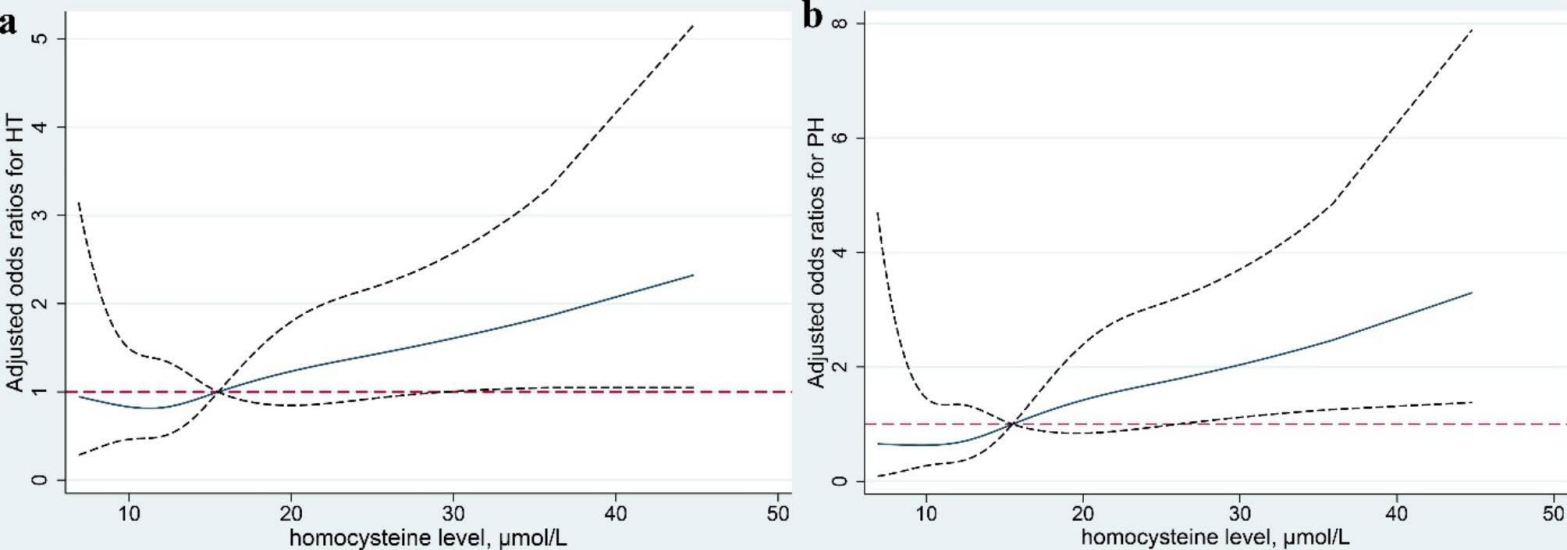
Adjusted dose-response associations between serum homocysteine (µmol/L) levels and the risk of HT (a) and PH (b). Serum homocysteine level was coded using a restricted cubic spline with four knots (at the 5th, 35th, 65th, and 95th percentiles of homocysteine). Solid lines stand for adjusted odds ratios (ORs) and dashed lines stand for their 95% confidence intervals. ORs were adjusted for sex, age, NIHSS on admission, atrial fibrillation, TOAST classification of stroke etiology, antiplatelet, interval time from admission to blood sampling, hypertension, diabetes mellitus, current smoking, blood creatine. Reference value is 15.5 µmol/L for homocysteine

The serum homocysteine levels were higher in patients with HT than those without (15.0(11.5–17.9) vs. 13.3(10.8–16.6) µmol/L, P = 0.048; Table 1). The higher homocysteine level group (≥ 15.5 µmol/L) were more likely to experience HT (19.0% vs. 10.2%, P = 0.011; Table 2). When homocysteine was regarded as a continuous variable, univariate and multivariate analysis showed increased serum homocysteine level was independently associated with an enhanced risk of HT (unadjusted OR 1.026, 95% CI 1.002–1.050, P = 0.034; adjusted OR 1.028, 95% CI 1.003–1.053, P = 0.027 in Model 1; and adjusted OR 1.029, 95%CI 1.003–1.055, P = 0.028 in Model 2; Table 3). Restricted cubic splines further showed the fluctuant change of adjusted OR along with the incremental change of homocysteine levels: homocysteine ≥ 15.5 µmol/L was associated with an increased magnitude of the association (adjusted OR for HT), which became significant when homocysteine ≥ 28µmol/L or so (Fig. 2a). Compared with the lower group, the higher group was more likely to have HT (adjusted OR 1.902, 95% CI 1.022–3.539, P = 0.042; Table 3).

**Table 3:** Univariate and multivariate logistic analysis of serum homocysteine level predicting for HT and PH

	Univariate analysis	Multivariate analysis, adjusted OR (95% CI), P value	
	unadjusted OR (95% CI), P value	Model 1	Model 2
**HT**			
Homocysteine (per 1 μmol/L increment)	1.026(1.002-1.050), 0.034	1.028(1.003-1.053), 0.027	1.029(1.003-1.055), 0.028
Homocysteine Levels			
<15.5μmol/L	Reference	Reference	Reference
≥15.5μmol/L	2.073(1.174-3.659), 0.012	2.194(1.201-4.007), 0.011	1.902(1.022-3.539), 0.042
**PH**			
Homocysteine (per 1 μmol/L increment)	1.037(1.011-1.064), 0.005	1.038(1.011-1.065), 0.006	1.041(1.013-1.070), 0.005
Homocysteine Levels			
<15.5μmol/L	Reference	Reference	Reference
≥15.5μmol/L	3.388(1.542-7.445), 0.002	3.443(1.503-7.887), 0.003	3.073(1.327-7.120), 0.009

HT, hemorrhagic transformation; PH, parenchyma hemorrhage; OR, odds ratio; CI, confidence interval

Model 1: adjusted for sex and age

Model 2: adjusted for sex, age, NIHSS on admission, atrial fibrillation and TOAST classification of stroke etiology, and interval time from admission to blood sampling

### Association between serum homocysteine and PH

Univariate analysis of baseline characteristics between AIS patients with and without PH showed a difference in serum homocysteine levels (16.5(12.5–21.1) vs. 13.3(10.8–16.4), P = 0.008; Table 1). Those in the higher group had a higher rate of PH (12.0% vs. 3.9%, P = 0.001; Table 2). After adjusting for sex, age, NIHSS on admission, atrial fibrillation, and TOAST classification, higher homocysteine levels were associated with an increased risk of PH (adjusted OR 1.041, 95% CI 1.013–1.070, P = 0.005; Table 3). Similarly, when homocysteine ≥ 15.5µmol/L, the magnitude of the association increased along with the incremental change of homocysteine levels (Fig. 2b). The adjusted OR for the higher group versus the lower group was 3.073 (95% CI 1.327–7.120, P = 0.009; Table 3).

### Subgroup analyses

**Fig. 3:**
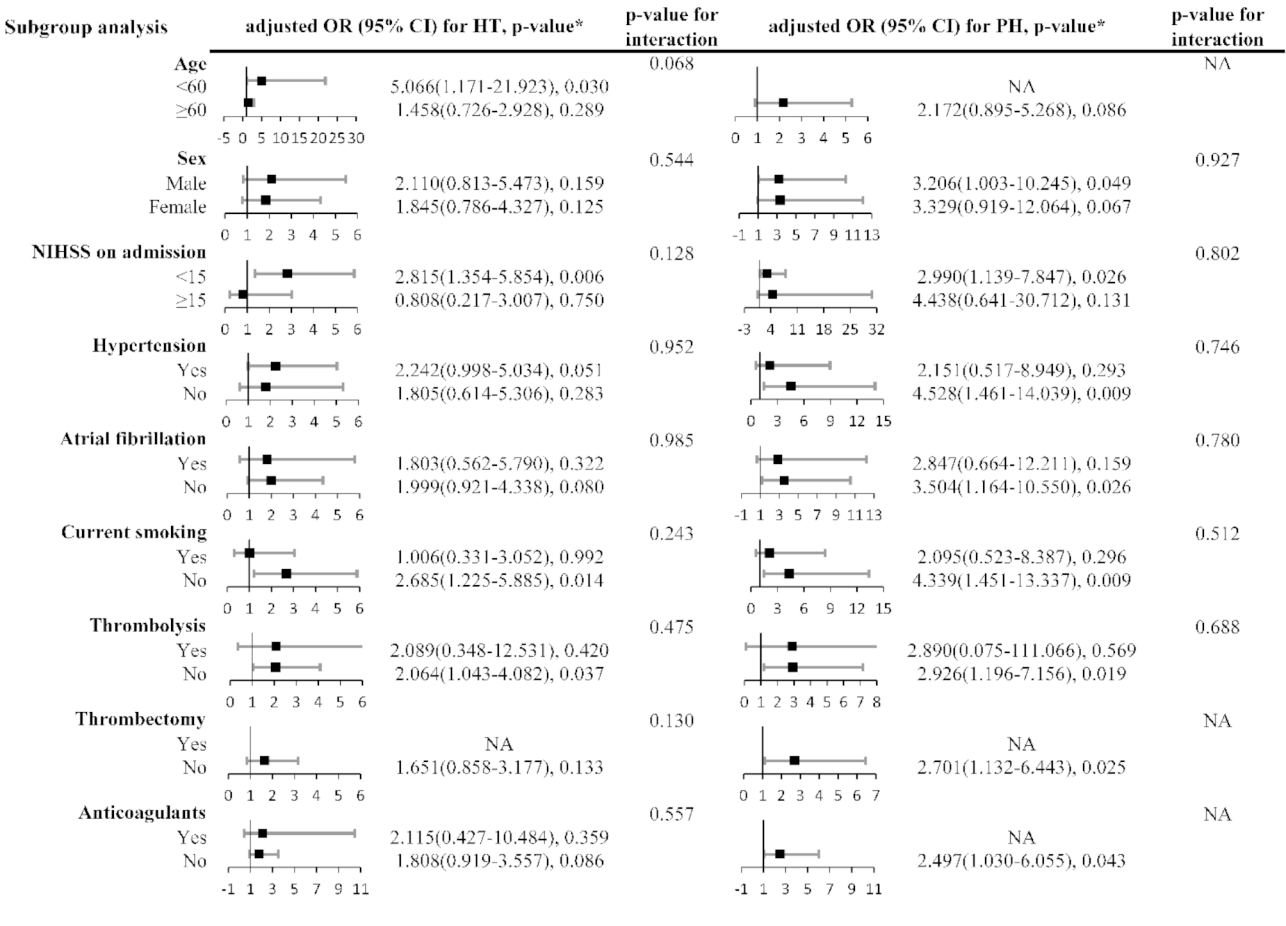
Stratified logistic regression analysis to identify variables that modify the correlations between higher homocysteine levels (≥ 15.5µmol/L) and HT and PH. P-value* for the corresponding OR of each subgroup, adjusted for the same variables as model 2 in Table 3 except for the stratified variable. Abbreviation: HT, hemorrhagic transformation; PH, parenchyma hemorrhage; OR, odds ratio; CI, confidence interval; NIHSS, National Institutes of Health Stroke Scale

We found the association between higher serum homocysteine levels (≥ 15.5µmol/L) and HT as well as PH was not altered by age, sex, NIHSS on admission, hypertension, atrial fibrillation, current smoking and medical treatments including thrombolysis and anticoagulation drugs (Fig. 3, all P > 0.05 for interactions). Subgroup analysis of patients without thrombolysis showed a significant relationship between higher serum homocysteine levels and HT (adjusted OR 2.064, 95% CI 1.043–4.082, P = 0.037) while this relationship was no longer significant in patients with thrombolysis (adjusted OR2.089, 95% CI 0.348–12.531, P = 0.420). Similar results were found in the associations between higher serum homocysteine and PH in those without thrombolysis (OR 2.926, 95% CI 1.196–7.156, P = 0.019). We also found increased serum homocysteine level (per 1 µmol/L) was correlated with HT (adjusted OR 1.042, 95%CI 1.010–1.076, P = 0.010) and PH (adjusted OR 1.053, 95%CI 1.017–1.091, P = 0.004) in patients without thrombolysis (Additional Table 2).

## Discussion

In this study, we found that higher serum homocysteine levels were independently associated with an enhanced risk of HT as well as PH when it was analyzed as a continuous and categorized variable, respectively. Limiting the analysis to patients without thrombolysis also showed a significant relationship between serum homocysteine levels and HT as well as PH. These findings highlight the need to take serum homocysteine into consideration when evaluating the risk of HT in AIS patients, especially those without thrombolysis.

Previous studies have reported that a large amount of homocysteine accumulation in the blood in some pathological conditions can increase the risk of cerebrovascular events [25]. More recently, it has been demonstrated that a higher level of plasma homocysteine (> 15.5 µmol/L) was related to an increased risk of recurrent ischemic stroke (adjusted HR 1.76, 95%CI 1.11–3.08)[12]. The relationship between serum homocysteine levels and HT after AIS, however, has been investigated in very few studies. Both Luo, et al [15] and Liu, et al [16] focused on the same topic but restricted to patients who received thrombolysis, with controversial conclusions. The former found hyperhomocysteinemia (defined as plasma homocysteine level ≥ 10 µmol/L) was not an independent risk factor for HT (OR 1.017, 95% CI 0.495–2.087), while the latter reported serum homocysteine level was an independent predictor for HT (OR 1.103, 95% CI 1.021–1.191). This may be due to different statistical methods used to analyze homocysteine as a continuous or categorized variable, the study participants, sample size or blood collection time. In the present study, by subgroup analysis, we found higher serum homocysteine levels were not significantly associated with an increased risk of HT in those with thrombolysis after adjusting for confounders, which is consistent with Luo’s study [15]. In addition, we found a significant relationship between serum homocysteine and HT in patients without thrombolysis after adjusting for confounders. This suggests that the role of homocysteine in the occurrence of HT in those who did not receive thrombolysis should also be considered. Large-sample sized studies are needed to verify the association between homocysteine and HT after thrombolysis in the future.

A dose-response association between serum homocysteine levels and the risk of HT as well as PH was also observed. The magnitude of the association between serum homocysteine levels and HT or PH greatly increased when homocysteine ≥ 15.5µmol/L, and became significant with the lower confidence interval above 1.0 when homocysteine was higher than around 28 µmol/L. These findings may provide a reference for cut-off value selection when exploring the relationship between homocysteine and HT in the future. This could also explain to some extent the controversial findings caused by different cut-off values of homocysteine among previous studies [15, 16, 26] .

The underlying mechanism as to why serum homocysteine levels are associated with HT have not been fully elucidated. However, experimental studies have provided some insight into the molecular mechanism of homocysteine destroying BBB structure and function, which allows extravasation of blood into brain. For example, Richard, et al. reported that homocysteine induced the disruption of BBB via N-methyl-D-aspartate (NMDA) receptor activation [27]. Furthermore, homocysteine can injure the structure of the BBB by antagonizing γ-aminobutyric acid A (GABA) receptors, increasing the activity of metalloproteinases [28], especially metalloproteinase-9, which have been shown to be crucial markers for BBB damage [29] and HT occurrence [30].

The results of this study should be interpreted carefully because of several limitations. First, this is a single-center hospital-based study with a small simple size, which may not represent the whole Chinese population and may be inappropriate to other populations worldwide. Larger, well-designed multicenter studies are needed to verify our findings. Second, a majority of patients without a blood homocysteine test were not enrolled in the analysis, which may cause selection bias. A baseline comparison between the included patients and the other participants enrolled from Chengdu Stroke Registry but not included in this study was performed, and there was no difference in any confounding variable (Additional Table 1). Third, serum homocysteine was tested and analyzed only once rather than continuously and dynamically monitored, and we had no data to examine the association between homocysteine changes and HT. Fourth, serum homocysteine can be influenced by nutritional status (e.g., Vitamin B12 levels), drugs (prescription and recreational), physical activity levels and genetic factors, which may affect the accuracy of our findings. Unfortunately, we could not collect detailed data about these factors.

## Conclusion

Higher serum homocysteine levels are associated with an increased risk of HT and PH in AIS patients, especially those without thrombolysis. Monitoring serum homocysteine may be conducive to determining individuals at a high risk of HT. Further investigations are needed to verify our findings and explore the potential molecular mechanism underlying the association between homocysteine and HT.

## Electronic supplementary material

Below is the link to the electronic supplementary material.

Supplementary Material 1

Supplementary Material 2

## Data Availability

The datasets used and/or analyzed in the current study are available from the corresponding author on reasonable request.

## Abbreviations

HT: Hemorrhagic transformation
AIS: Acute ischemic stroke
PH: Parenchymal hematoma
BBB: Blood brain barrier
CT: Computed tomography
MRI: Magnetic resonance imaging
NIHSS: National Institutes of Health Stroke Scale
TOAST: The Trial of ORG 10172 in Acute Stroke Treatment
ORs: Odds ratios
CI: Confidence interval
